# Mapeamento Potencial de Superfície Corporal durante a Despolarização Ventricular em Ratos Após Exercício Exaustivo Agudo

**DOI:** 10.36660/abc.20211058

**Published:** 2022-09-06

**Authors:** Alexey G. Ivonin, Svetlana L. Smirnova, Irina M. Roshchevskaya

**Affiliations:** 1 Department of Comparative Cardiology Komi Scientific Centre of the Ural Branch Russian Academy of Sciences Syktyvkar Federação Russa Department of Comparative Cardiology – Komi Scientific Centre of the Ural Branch of the Russian Academy of Sciences , Syktyvkar – Federação Russa; 2 Laboratory of Pharmacological Screening Research Zakusov Institute of Pharmacology Moscow Federação Russa Laboratory of Pharmacological Screening – Research Zakusov Institute of Pharmacology , Moscow – Federação Russa

**Keywords:** Ratos, Ventrículos do Coração, Miocárdio, Exercícios, Agudo, Mapeamento de Potencial de Superfície Corporal

## Abstract

**Fundamento:**

O exercício físico exaustivo pode causar alterações significantes nas propriedades elétricas do miocárdio.

**Objetivo:**

Avaliar, através do mapeamento potencial de superfície corporal, a atividade elétrica do coração de ratos durante a despolarização ventricular após exercício exaustivo agudo.

**Métodos:**

Ratos machos com doze semanas de idade foram submetidos a exercício agudo em esteira a 36 m/min até a exaustão. Eletrocardiogramas unipolares (ECGs) da superfície do tronco foram registrados em ratos anestesiados com zoletil três a cinco dias antes (Pré-Ex), 5 e 10 minutos após exercício exaustivo (Pós-Ex 5 e Pós-Ex 10, respectivamente) simultaneamente com ECGs nas derivações dos membros. Os mapas potenciais de superfície corporal instantâneos (BSPMs, *body surface potential maps* ) foram analisados durante a despolarização ventricular. Os valores de p <0,05 foram considerados estatisticamente significantes.

**Resultados:**

Comparado com o Pré-Ex, uma conclusão precoce da segunda inversão de distribuições de potencial, uma conclusão precoce da despolarização ventricular, bem como uma diminuição na duração da fase média e a duração total da despolarização ventricular nos BSPMs foram reveladas no Pós-Ex5. Além disso, em comparação com o Pré-Ex, um aumento na amplitude do extremo negativo do BSPM no pico da onda R no ECG na derivação II (pico RII) e uma diminuição na amplitude do extremo negativo do BSPM a 3 e 4 ms após o pico RII foram demonstrados no Pós-Ex 5. No Pós-Ex 10, os parâmetros dos BSPMs não diferiram daqueles do Pré-Ex.

**Conclusão:**

Em ratos, o exercício exaustivo agudo causa alterações reversíveis nas características temporais e de amplitude dos BSPMs durante a despolarização ventricular, provavelmente relacionadas a alterações na excitação da massa principal do miocárdio ventricular.

## Introdução

Os efeitos benéficos do exercício físico regular na saúde pública, incluindo a redução do risco de doenças cardiovasculares, estão bem estabelecidos. ^1 , 2^ Entretanto, o excesso de exercício pode causar danos ao organismo, principalmente ao coração. Vários estudos demonstraram uma deterioração transitória da função cardíaca após episódios agudos de exercícios de resistência prolongados em indivíduos aparentemente saudáveis. ^3 , 4^ O excesso de exercício físico pode ser um gatilho para arritmias ventriculares malignas, infarto agudo do miocárdio e morte súbita cardíaca em pessoas suscetíveis. ^5^

A pesquisa sobre o impacto do exercício exaustivo no sistema cardiovascular é essencial. Mas, em humanos, é difícil determinar objetivamente a exaustão em condições de laboratório, uma vez que ela é medida pela incapacidade do indivíduo de manter o regime de exercícios por esforço volitivo. ^6 , 7^ Nesse sentido, a vantagem dos modelos animais (por exemplo, ratos, camundongos) na avaliação de respostas fisiológicas a exercícios exaustivos é que existem critérios objetivos para a definição de exaustão, como a incapacidade de um animal continuar correndo apesar dos estímulos externos durante um teste de corrida em esteira ^8 , 9^ ou afundar em uma piscina durante um teste de natação forçada. ^10^

Numerosos estudos em animais de laboratório mostraram danos ao tecido miocárdico e comprometimento da função cardíaca associados à apoptose de cardiomiócitos, estresse oxidativo e respostas inflamatórias após episódios agudos de exercício exaustivo. ^11 - 13^ É relatado que o exercício exaustivo agudo causa alterações nos padrões eletrocardiográficos em ratos, sugerindo alterações na despolarização ventricular. ^14 , 15^

O mapeamento potencial da superfície corporal com base em um registro simultâneo de potenciais gerados pelo coração, a partir de vários locais, em toda a superfície do tronco, fornece mais informações fisiológicas e diagnósticas sobre eventos elétricos no miocárdio em comparação com a eletrocardiografia convencional. ^16 - 18^ Ao utilizar o mapeamento potencial da superfície corporal, o presente estudo teve como objetivo avaliar a atividade elétrica do coração em ratos de laboratório durante a despolarização ventricular após exercício exaustivo agudo.

## Métodos

### Animais experimentais

Ratos albinos machos não consanguíneos de doze semanas de idade (n=24), pesando entre 200 e 300 g, foram adquiridos da *Stolbovaya Branch of the Scientific Center for Biomedical Technologies of the Federal Medical-Biological Agency* (Federação Russa). Os ratos foram alojados em gaiolas grupais em uma sala com temperatura controlada (22 ± 2°C) sob um ciclo claro/escuro de 12 horas e alimentados com ração comercial padrão para roedores e água *ad libitum* . Todos os procedimentos e protocolos experimentais foram realizados de acordo com o Guia para o Cuidado e Uso de Animais de Laboratório publicado pelo *National Institutes of Health* (Publicação NIH No. 85-23, revisado em 1996) e aprovado pelo Comitê de Ética da *Komi Science Centre of the Ural Branch of the Russian Academy of Sciences* (Syktyvkar, Federação Russa).

### Protocolo de exercício

Uma esteira motorizada para roedores (Panlab / Harvard Apparatus, Espanha) foi utilizada para criar o exercício agudo exaustivo para ratos. Pulsos elétricos leves (0,5 mA) da grade eletrificada na parte traseira da esteira motivaram os animais a se exercitarem. Antes do experimento, os ratos foram acostumados ao exercício em esteira por três dias consecutivos (10 min/dia, 12-36 m/min, 0° de inclinação). Apenas alguns ratos, capazes de correr em modo adaptativo, foram selecionados para o estudo mais aprofundado. No dia do teste de exercício, os ratos correram a uma velocidade de 36 m/min e inclinação de 0° até a exaustão. A exaustão foi definida como o ponto em que o rato não conseguia mais correr, apesar de ser empurrado contra a grade de choque pela esteira em movimento. ^8^ O tempo de corrida até a exaustão foi calculado com o software Sedacom versão 2.0 (Panlab / Harvard Apparatus, Espanha).

### Eletrocardiografia convencional e mapeamento potencial de superfície corporal

**Figura 1:**
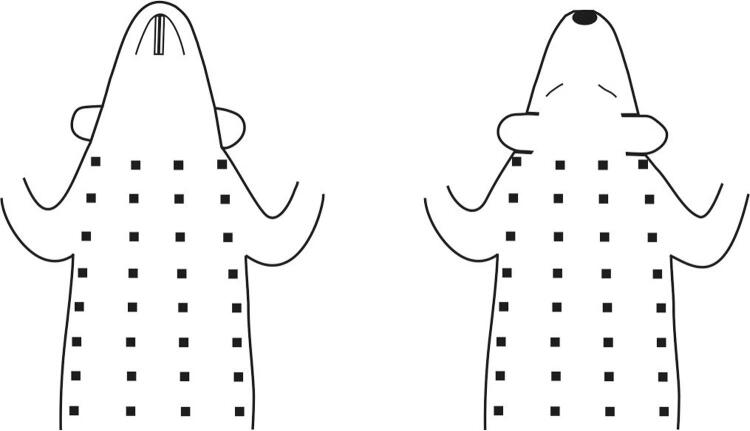
Posições dos eletrodos na superfície corporal do rato utilizando 64 derivações unipolares no tronco. Quatro fileiras de eletrodos de agulha foram colocados na superfície ventral e quatro fileiras na superfície dorsal do corpo (oito eletrodos em cada fileira). Ao usar 32 derivações unipolares do tronco, o número de fileiras de eletrodos nas superfícies ventral e dorsal do corpo diminuiu duas vezes.

O registro da atividade elétrica cardíaca foi realizado três a cinco dias antes (Pré-Ex), 5 e 10 minutos após (Pós-Ex 5 e Pós-Ex 10, respectivamente) o exercício agudo exaustivo. Os ratos foram anestesiados com Zoletil (combinação de tiletamina/zolazepam, Virbac, França) na dose de 3,5 mg/100 g, i.m. e colocados em decúbito dorsal sobre uma almofada de aquecimento para manter a temperatura corporal aproximadamente em 37°C. Eletrocardiogramas unipolares (ECGs) foram registrados a partir de eletrodos agulha subcutâneos, distribuídos uniformemente ao redor do tronco, desde o nível da junção cervicotorácica até as margens costais inferiores ( Figura 1 ). Foram utilizados 64 eletrodos no Pré-Ex e 32 eletrodos no Pós-Ex 5 e Pós-Ex 10. Simultaneamente com ECGs unipolares da superfície do tronco, ECGs foram registrados em derivações de membros bipolares. O terminal central Wilson serviu como referência para derivações unipolares do tronco. Os dados foram adquiridos utilizando um sistema de computador multicanal (largura de banda de 0,05-1000 Hz, taxa de amostragem de 4000 Hz e precisão de 16 bits). Os ratos tiveram um período de três a cinco dias para se recuperar da anestesia antes do exercício exaustivo. Para registro dos potenciais cardíacos pós-exercício, os ratos foram anestesiados imediatamente após a exaustão.

A duração dos intervalos R-R e os parâmetros de despolarização ventricular (duração do QRS, duração e amplitude das ondas R e S, soma das amplitudes das ondas R e S) foram analisados no ECG na derivação II do membro (ECG _II_ ). Os intervalos R-R foram usados para calcular a frequência cardíaca (FC). Com base nos ECGs unipolares, foram construídos mapas equipotenciais (isopotenciais) instantâneos da superfície corporal, refletindo a distribuição dos potenciais cardíacos em cada instante de despolarização ventricular em um padrão plano da superfície torácica alinhado a um plano retangular. Os mapas potenciais de superfície corporal (BSPMs, *body surface potential maps* ) analisaram a localização espacial e as trajetórias de deslocamento das áreas e extremos de potenciais positivos e negativos na superfície do tórax, as amplitudes dos extremos em instantes sequenciais de tempo durante a despolarização ventricular, as amplitudes máximas dos extremos para toda a despolarização ventricular e o momento em que os extremos atingiram amplitudes máximas. As características temporais dos BSPMs foram apresentadas em ms em relação ao pico da onda RII (até o pico R _II_ – com sinal negativo).

De acordo com a dinâmica espaço-temporal da distribuição do potencial de superfície corporal, as seguintes fases de despolarização ventricular foram diferenciadas: ^19^

1. Fase inicial – desde a formação de um padrão de distribuição potencial cardíaco, correspondente à despolarização ventricular, até a conclusão da primeira inversão de áreas potenciais positivas e negativas.
2. Fase média – desde a conclusão da primeira inversão até a conclusão da segunda inversão de áreas potenciais.
3. Fase terminal – desde a conclusão da segunda inversão de áreas potenciais até o desaparecimento do padrão de distribuição potencial correspondente à despolarização ventricular.

Para cada rato, as características do ECG _II_ e BSPMs foram determinadas de três a cinco batimentos no Pré-Ex, Pós-Ex 5 e Pós-Ex 10.

### Análise estatística

A análise estatística foi realizada pelo pacote de *software* Statistica (versão 10.0, StatSoft, Tulsa, OK, EUA). A normalidade dos dados contínuos foi verificada pelo teste de Shapiro-Wilk. As variáveis com distribuição normal foram expressas como média ± desvio padrão e as variáveis com distribuição não-normal foram apresentadas como mediana, primeiro e terceiro quartis. Para os dados com distribuição normal, foi utilizada a análise de variância (ANOVA) de medidas repetidas, seguida do teste de comparação múltipla de Dunnett como análise *post-hoc* . Quando os dados não estavam distribuídos normalmente, foi realizado o teste não-paramétrico de Friedman, seguido do teste de Wilcoxon com ajuste de Bonferroni. A significância estatística foi estabelecida em nível alfa de 0,05, exceto para o teste de Wilcoxon, no qual o nível alfa foi ajustado para 0,025 (de acordo com o número de comparações pareadas) para evitar erro tipo I. O tamanho da amostra foi determinado por conveniência. Foram considerados dados ^20^ indicando que até 10% dos ratos de fornecedores comerciais se recusam a correr em esteira e precisam ser eliminados dos estudos de exercícios.

## Resultados

### Desempenho no exercício

Dos 24 ratos utilizados em sessões de familiarização de corrida, 20 foram selecionados para o exercício exaustivo agudo de acordo com sua capacidade de corrida. Nesses ratos, o tempo de corrida até a exaustão foi de 19,5 ± 5,6 min.

### Parâmetros convencionais de ECG

A Tabela 1 mostra os achados do ECG _II_ . Não houve alterações estatisticamente significantes nos valores de FC após exercício exaustivo em esteira. Comparados aos valores do Pré-Ex, a duração do QRS _II_ e a duração da onda R _II_ foram menores no Pós-Ex 5. Comparados com os do Pré-Ex, a amplitude da onda S _II_ e a soma das amplitudes das ondas R _II_ e S _II_ foram maiores no Pós-Ex 5 e Pós-Ex 10. Não houve mudanças significantes na duração da onda S _II_ ou na amplitude da onda R _II_ após corrida exaustiva em esteira.

**Tabela 1:** Parâmetros ECG II antes e após exercício exaustivo agudo em esteira

	Pré-Ex	Pós-Ex 5	Pós-Ex 10
FC, bpm	480,3 ± 23,3	484,5 ± 25,2	490,7 ± 28,8
Duração do QRS _II_ , ms	16,0 ± 1,1	15,3 ± 1,2*	15,8 ± 1,7
Duração da onda R _II_ , ms	9,7 ± 1,0	9,1 ± 0,9*	9,4 ± 1,0
Duração da onda S _II_ , ms	6,3 ± 1,4	6,2 ± 1,5	6,3 ± 1,7
Amplitude da onda R _II_ , mV	0,62 ± 0,16	0,65 ± 0,15	0,63 ± 0,14
Amplitude da onda S _II_ , mV	-0,28 ± 0,15	-0,35 ± 0,18*	-0,35 ± 0,19*
Soma das amplitudes das ondas R _II_ e S _II_ , mV	0,90 ± 0,19	0,99 ± 0,20*	0,98±0,20*

Os dados são expressos como média ± desvio padrão (n = 20). Pré-Ex: antes do exercício exaustivo; Pós-Ex 5: 5 minutos após exercício exaustivo; Pós-Ex 10: 10 minutos após exercício exaustivo. FC: frequência cardíaca. ANOVA de medidas repetidas e teste post-hoc de Dunnett; *p < 0,05 vs. Pré-Ex.

### Padrão espacial dos BSPMs

**Figura 2:**
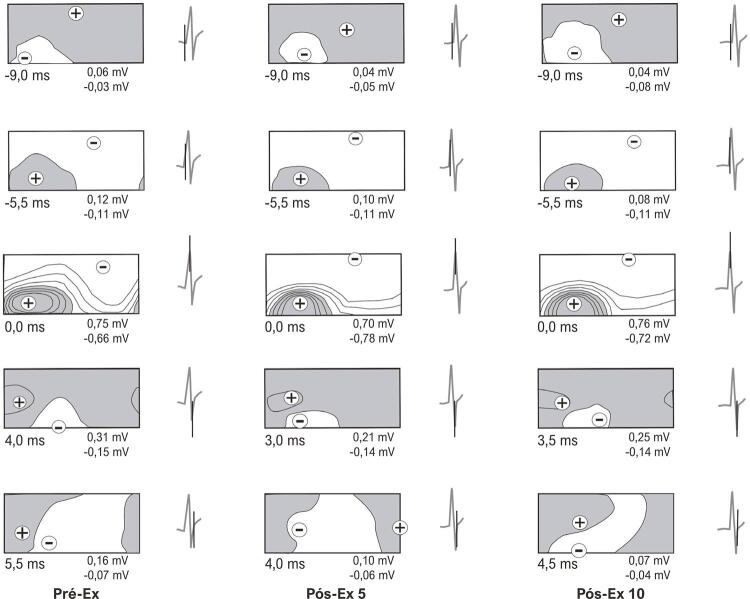
Mapas equipotenciais da superfície corporal durante a despolarização ventricular antes e após exercício exaustivo em esteira do mesmo rato. As áreas de potenciais positivos (preenchidas) e negativos são mostradas. Os sinais, mais e menos, denotam a localização dos extremos positivo e negativo, respectivamente. Abaixo de cada mapa, são mostrados o tempo relativo ao pico da onda RII e as amplitudes dos extremos positivo e negativo. Perto de cada mapa, o ECGII é mostrado com um marcador de tempo (linha vertical). O espaçamento de contorno isopotencial é de 0,2 mV. Em cada mapa, a parte esquerda representa a superfície ventral do corpo e a direita representa a superfície dorsal do corpo. Pré-Ex: antes do exercício exaustivo; Pós-Ex 5: 5 minutos após exercício exaustivo; Pós-Ex 10: 10 minutos após exercício exaustivo.

Antes do exercício exaustivo, o padrão de distribuição potencial de superfície corporal, correspondente ao início da despolarização ventricular, foi observado antes do aparecimento do complexo QRS no ECG _II_ ( Figura 2 , instante Pré-Ex,–9 ms). Nesse caso, a área dos potenciais cardíacos positivos cobria a parte cranial do tórax ventral e todo o dorso do tórax, com o extremo positivo localizado principalmente no terço cranial do tórax lateral esquerdo. A área de potenciais negativos e o extremo negativo localizavam-se na parte caudal do tórax ventral. Durante a subida da onda R _II_ , ocorreu a primeira inversão das distribuições de potencial nos BSPMs e, como resultado, as áreas positivas e negativas mudaram suas posições relativas ( Figura 2 , Pré-Ex, instante –5,5 ms). Ao término da primeira inversão, o extremo positivo localizava-se de forma caudal no tórax ventral, e o extremo negativo localizava-se cranialmente, mais frequentemente no dorso. No instante do pico da onda R _II_ , a localização das áreas e extremos nos BSPMs permaneceu essencialmente inalterada ( Figura 2 , Pré-Ex, instante 0 ms). Durante a descida das ondas R _II_ e S _II_ , a segunda inversão das distribuições de potenciais foi observada nos BSPMs e, como consequência, a área de potenciais negativos se localizou na parte caudal do tórax, lateralmente ou ventralmente à esquerda, enquanto a área de potenciais positivos ocupou a superfície torácica remanescente ( Figura 2 , Pré-Ex, instante 4,0 ms). Ao completar a segunda inversão, o extremo positivo se deslocou cranialmente e lateralmente à direita, com o extremo negativo sendo localizado caudalmente no lado esquerdo do tórax. Durante a transição da onda S _II_ para a onda T _II_ , foi registrado um padrão nos BSPMs de uma posição instável das áreas positiva e negativa, que indicava a conclusão da despolarização ventricular ( Figura 2 , Pré-Ex, instante 5,5 ms). Após o exercício exaustivo, as distribuições potenciais espaciais durante a despolarização ventricular foram bastante semelhantes às do Pré-Ex ( Figura 2 , Pós-Ex 5 e Pós-Ex 10).

### Características temporais dos BSPMs

**Figura 3:**
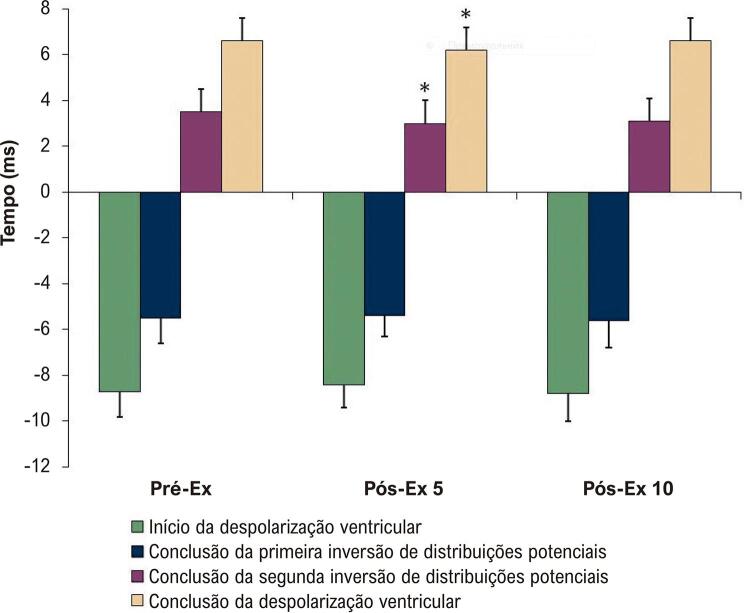
Parâmetros temporais dos BSPMs durante a despolarização ventricular, antes e após exercício exaustivo em esteira. O tempo é mostrado em relação ao pico da onda RII. Os dados são expressos como média ± desvio padrão (n = 20). Pré-Ex: antes do exercício exaustivo; Pós-Ex 5: 5 minutos após exercício exaustivo; Pós-Ex 10: 10 minutos após exercício exaustivo. ANOVA de medidas repetidas e teste post-hoc de Dunnett; *p < 0,05 vs. Pré-Ex.

Não houve alterações significantes no momento do início da despolarização ventricular ou no momento da conclusão da primeira inversão de distribuições potenciais nos BSPMs após exercício exaustivo em esteira ( Figura 3 ). Comparada com a do Pré-Ex, a segunda inversão de distribuições potenciais nos BSPMs foi concluída significantemente mais cedo no Pós-Ex 5 ( Figura 3 ). Além disso, em comparação com o Pré-Ex, a despolarização ventricular nos BSPMs foi concluída significantemente mais cedo no Pós-Ex 5 ( Figura 3 ).

**Figura 4:**
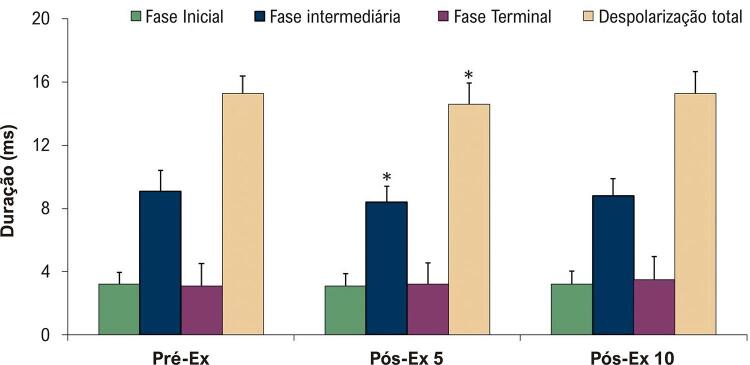
Duração das fases individuais e duração total da despolarização ventricular nos BSPMs antes e após exercício agudo exaustivo em esteira. Os dados são expressos como média ± desvio padrão (n = 20). Pré-Ex: antes do exercício exaustivo; Pós-Ex 5: 5 minutos após exercício exaustivo; Pós-Ex 10: 10 minutos após exercício exaustivo. ANOVA de medidas repetidas e teste post-hoc de Dunnett; *p < 0,05 vs. Pré-Ex.

Não foram observadas alterações significantes na duração das fases inicial e terminal da despolarização ventricular nos BSPMs após corrida exaustiva em esteira ( Figura 4 ). Em comparação com as do Pré-Ex, a duração da fase média e a duração total da despolarização ventricular diminuíram no Pós-Ex 5 ( Figura 4 ).

### Características dos extremos de BSPM

**Figura 5:**
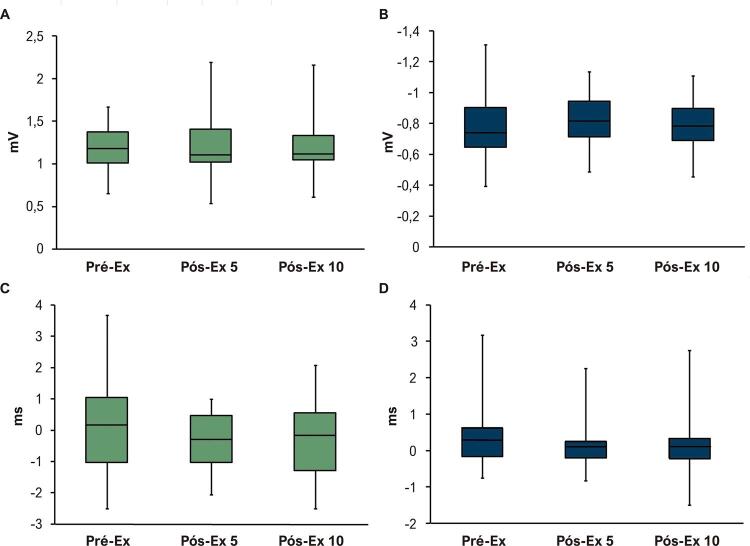
Amplitudes máximas de extremos positivo (A) e negativo (B) nos BSPMs durante despolarização e o momento em que os extremos positivo e negativo atingiram amplitudes máximas (C e D, respectivamente) antes e após o exercício exaustivo agudo em esteira. O tempo é mostrado em relação ao pico da onda RII. Os dados são expressos em mediana, primeiro e terceiro quartis, valores mínimo e máximo (n = 20). Pré-Ex: antes do exercício exaustivo; Pós-Ex 5: 5 minutos após exercício exaustivo; Pós-Ex 10: 10 minutos após exercício exaustivo. Teste de Friedman, p > 0,05.

Durante a despolarização ventricular, as amplitudes dos extremos positivo e negativo dos BSPMs pré e pós exercício exaustivo em esteira aumentaram progressivamente, atingindo valores máximos próximos ao pico da onda R _II_ , depois diminuíram ( Figura 2 , Tabela 2 ). Não houve alterações significantes nas amplitudes máximas dos extremos dos BSPMs durante a despolarização ventricular, nem no momento em que os extremos atingiram amplitudes máximas após exercício exaustivo agudo ( Figura 5 ).

**Tabela 2:** Amplitudes dos extremos do BSPM em diferentes instantes de tempo durante a despolarização ventricular antes e após exercício exaustivo agudo em esteira

		Momento, ms									
		-5	-4	-3	-2	-1	0	1	2	3	4
Extremo positivo, mV	Pré-Ex	0,19 (0,06; 0,31)	0,44 (0,19; 0,54)	0,67 (0,41; 0,81)	0,89 (0,80; 1,09)	1,06 (0,90; 1,20)	0,92 (0,79; 1,16)	0,74 (0,54; 0,91)	0,45 (0,17; 0,79)	0,41 (0,29; 0,63)	0,4 (0,33; 0,52)
	Pós-Ex 5	0,14 (0,05; 0,24)	0,34 (0,19; 0,46)	0,57 (0,43; 0,73)	0,88 (0,67; 1,05)	1,02 (0,78; 1,20)	0,95 (0,77; 1,15)	0,76 (0,39; 0,9)	0,33 (0,22; 0,59)	0,44 (0,19; 0,59)	0,34 (0,20; 0,46)
	Pós -Ex 10	0,19 (0,06; 0,28)	0,41 (0,19; 0,50)	0,67 (0,44; 0,77)	0,84 (0,56;1,02)	0,95 (0,75; 1,16)	0,95 (0,76; 1,15)	0,61 (0,28; 0,91)	0,39 (0,18; 0,57)	0,41 (0,21; 0,58)	0,4 (0,2; 0,53)
Extremo negativo, mV	Pré-Ex	-0,14 (-0,11; -0,21)	-0,28 (-0,16; -0,35)	-0,40 (-0,26; -0,52)	-0,58 (-0,38; -0,64)	-0,64 (-0,51; -0,73)	-0,71 (-0,62; -0,85)	-0,58 (-0,49; -0,74)	-0,42 (-0,29; -0,56)	-0,28 (-0,20; -0,40)	-0,16 (-0,09; -0,27)
	Pós -Ex 5	-0,14 (-0,07; -0,19)	-0,26 (-0,18; -0,35)	-0,42 (-0,27; -0,50)	-0,57 (-0,41;-0,67)	-0,65 (-0,58; -0,78)	-0,77 (-0,68; -0,92)*	-0,65 (-0,44; -0,78)	-0,39 (-0,22; -0,44)	-0,21 (-0,14; -0,30)*	-0,08 (-0,05; -0,189)*
	Pós -Ex 10	-0,16 (-0,08; -0,20)	-0,29 (-0,17; -0,35)	-0,45 (-0,30; -0,53)	-0,60 (-0,40; -0,69)	-0,66 (-0,55; -0,75)	-0,75 (-0,60; -0,90)	-0,67 (-0,45; -0,73)	-0,39 (-0,20; -0,46)	-0,23 (-0,13; -0,36)	-0,12 (-0,06; -0,23)

O tempo é mostrado em relação ao pico da onda RII. Os dados são expressos como mediana, primeiro e terceiro quartis (n = 20). Pré-Ex: antes do exercício exaustivo; Pós-Ex 5: 5 minutos após exercício exaustivo; Pós-Ex 10: 10 minutos após exercício exaustivo. Teste de Wilcoxon com ajuste de Bonferroni; *p < 0,025 vs. Pré-Ex no mesmo tempo.

As amplitudes dos extremos de BSPM em diferentes instantes de tempo durante a despolarização ventricular são mostradas na Tabela 2 . Devido às variações individuais nos tempos de início e término da despolarização ventricular nos BSPMs, o intervalo de tempo correspondente à despolarização dos ventrículos em todos os animais estudados (ou seja, de -5 a 4 ms em relação ao pico R _II_ ) foi analisado. Em cada momento analisado do intervalo de tempo escolhido, não foram observadas alterações significantes na amplitude do extremo positivo do BSPM após exercício exaustivo em esteira. No momento do pico R _II_ , a amplitude do extremo negativo do BSPM no Pós-Ex 5 foi maior em comparação com o Pré-Ex ( Tabela 2 , momento 0 ms). Além disso, a 3 e 4 ms após o pico R _II_ , as amplitudes do extremo negativo do BSPM no Pós-Ex 5 foram menores em comparação com as do Pré-Ex ( Tabela 2 , momentos 3 e 4 ms).

## Discussão

No presente estudo, avaliamos a atividade elétrica do coração durante a despolarização ventricular após exercício exaustivo agudo em esteira em ratos previamente não treinados, utilizando mapeamento potencial de superfície corporal em combinação com eletrocardiografia convencional.

A resposta cardiovascular imediata ao exercício forçado em esteira em roedores é o aumento da FC ^21 , 22^ garantindo o aumento do débito cardíaco. Em ratos Wistar, a aceleração da FC, durante a corrida em esteira, é determinada predominantemente pela atividade do nervo autonômico cardíaco, especialmente pelo aumento do fluxo simpático. ^23^ A diminuição da FC, após a cessação do exercício físico, é provavelmente a manifestação da reativação vagal. ^24^ Estudos anteriores em ratos não treinados demonstraram o declínio da FC para o nível inicial aos 10 minutos após corrida exaustiva aguda em esteira. ^25^ No presente estudo, em ambas as situações após exercício exaustivo (Pós-Ex 5 e Pós-Ex 10), os valores da FC foram quase iguais aos da linha basal (Pré-Ex). Em nossa opinião, esses resultados podem ser explicados por uma diminuição da FC para o nível pré-exercício nos primeiros 5 minutos após a corrida até a exaustão.

Em seres humanos saudáveis, a resposta do ECG ao exercício físico consiste tanto em uma diminuição na duração do QRS ou em nenhuma alteração na duração do QRS. ^26 , 27^ O encurtamento da duração do QRS com o exercício em indivíduos saudáveis é atribuído ao aumento da velocidade de condução intraventricular, devido ao aumento do tônus adrenérgico. ^26^ Enquanto isso, o prolongamento do QRS com o exercício é considerado um achado anormal do ECG e pode servir como marcador de isquemia miocárdica. ^28^ Foi observado o prolongamento significante da duração do QRS, acompanhado pelo comprometimento da capacidade funcional cardíaca em ratos Sprague-Dawley saudáveis, que foram submetidos a exercício exaustivo aguda em esteira, em comparação com seus controles sedentários. ^15^ Encontramos uma diminuição na duração do QRS _II_ e da duração da onda R _II_ no Pós-Ex em comparação com a do Pré-Ex, enquanto no Pós-Ex 10, esses parâmetros retornaram ao nível próximo ao Pré-Ex. É geralmente aceito que, em humanos, a onda R nas derivações de membros no ECG representa a despolarização dos ventrículos esquerdo e direito, e a onda S reflete predominantemente a excitação das partes basais dos ventrículos. ^29^ A gênese das ondas R e S em ECGs em ratos é semelhante à de humanos. ^30^ Assim, nossos resultados sugerem que as mudanças na duração do QRS após exercício exaustivo agudo em ratos estavam relacionadas a uma diminuição transitória na duração da excitação da maior parte do miocárdio ventricular.

Em humanos aparentemente saudáveis, uma diminuição da amplitude da onda R, com ou sem aumento da amplitude da onda S, é observada nos ECGs no exercício máximo. ^31 , 32^ A redução na amplitude da onda R, com exercício extenuante, é atribuída a uma diminuição do volume diastólico final ventricular, ^27^ bem como a alterações na ativação elétrica cardíaca, ^31^ enquanto presume-se que o aumento na amplitude da onda S está relacionado a deslocamentos do eixo elétrico ou alterações de condução ventricular. ^33^ Nesse estudo, a amplitude da onda R _II_ não se alterou após a corrida exaustiva em esteira. Entretanto, em comparação com o Pré-Ex, a amplitude da onda S _II_ aumentou no Pós-Ex 5 e Pós-Ex 10. Uma vez que a soma das amplitudes das ondas R _II_ e S _II_ também aumentou no Pós-Ex 5 e Pós-Ex 10, as variações na amplitude da onda S _II_ após exercício exaustivo provavelmente não foram resultado de um desvio do eixo QRS. Devido à falta de um segmento ST isoelétrico em ratos, o aumento da amplitude da onda S no ECG de ratos é considerado como depressão do segmento ST e é interpretado como um sinal de isquemia miocárdica. ^34 , 35^ Assim, no presente estudo, o aumento da amplitude da onda S _II_ em ratos exaustos pode ser causado por alterações isquêmicas induzidas pelo exercício nos ventrículos cardíacos. No entanto, mais estudos são necessários para confirmar essa suposição.

Mudanças na distribuição espacial dos potenciais cardíacos na superfície do corpo, durante a despolarização ventricular, são indicadores de alterações na direção das frentes de onda de excitação nos ventrículos cardíacos. ^16 , 36^ Miller et al. ^37^ observaram mudanças significantes na trajetória de migração do extremo negativo, bem como o aparecimento dos extremos anteriores positivos adicionais nos BSPMs durante o complexo QRS, no exercício máximo em indivíduos saudáveis, o que eles consideraram como sendo devido a um atraso na ativação da parede livre do ventrículo esquerdo em relação ao início da excitação no ventrículo direito e septo intraventricular.

Takala et al. ^38^ não revelaram alterações nos padrões espaciais dos BSPMs do intervalo QRS-tempo após exercício máximo em indivíduos saudáveis. No presente estudo, os padrões espaciais do BSPM durante a despolarização ventricular foram semelhantes aos observados anteriormente em ratos Wistar saudáveis. ^39^ As localizações das áreas e extremos, e sua dinâmica nos BSPMs em Pós-Ex 5 e Pós-Ex 10 foram quase idênticas àquelas no Pre-Ex, o que sugeriu que a direção principal da onda de ativação em ventrículos de ratos não mudou muito com o exercício exaustivo agudo em esteira.

Em ratos, a primeira inversão das distribuições de potencial nos BSPMs, durante a despolarização ventricular, é causada por um rompimento da onda de excitação no subepicárdio, tanto da base do ventrículo direito quanto do ápice do ventrículo esquerdo, e a segunda inversão resultou da mudança na direção da onda de ativação em direção à base do ventrículo esquerdo e ao cone excretor da aorta. ^19^ Durante a fase inicial da despolarização ventricular nos BSPMs, a onda de excitação, em ratos, propaga-se pelo sistema condutor e depois move-se através do miocárdio em direção endo-epicárdica. Durante as fases média e terminal da despolarização ventricular nos BSPMs, há excitação da massa principal do miocárdio ventricular e da base do ventrículo esquerdo, respectivamente. ^19^ Nesse estudo, no Pós-Ex 5, em comparação com o Pré-Ex, foi demonstrado uma conclusão precoce da segunda inversão de distribuições potenciais, uma conclusão precoce da despolarização ventricular, bem como uma diminuição na duração da fase média e da duração total da despolarização ventricular nos BSPMs, o que parece ser resultado de uma diminuição na duração da ativação da massa principal do miocárdio ventricular. No Pós-Ex 10, os parâmetros temporais dos BSPMs não diferiram do Pré-Ex. Assim, no presente estudo, as mudanças nas características temporais do BSPM, subsequentes ao exercício exaustivo agudo em esteira, foram causadas por uma redução reversível na duração da fase média da despolarização ventricular, enquanto as durações das fases inicial e terminal permaneceram essencialmente inalteradas. Comparado com o eletrocardiograma convencional, o mapeamento potencial de superfície corporal permitiu a identificação mais precisa do estágio de despolarização ventricular, cuja duração se alterou consideravelmente em consequência do exercício exaustivo de ratos em esteira.

Em relação ao efeito do exercício extenuante nos parâmetros de amplitude do BSPM, Mirvis ^40^ descreveu uma redução na amplitude do extremo positivo e um aumento na amplitude do extremo negativo nos mapas isopotenciais da superfície anterior do tórax em diferentes pontos do complexo QRS durante o exercício submáximo em indivíduos saudáveis. Outros autores ^37^ revelaram a diminuição da amplitude máxima do extremo positivo do BSPM durante o QRS, com exercício máximo em voluntários saudáveis, o que eles supuseram ser resultado das mudanças nas frentes de onda de ativação na parede do ventrículo esquerdo. Em nosso estudo, as amplitudes máximas dos extremos positivo e negativo do BSPM durante a despolarização ventricular e o tempo em que os extremos atingiram as amplitudes máximas não se alteraram significantemente após o exercício exaustivo em esteira. Enquanto isso, em comparação com o Pré-Ex, a amplitude do extremo negativo do BSPM no instante do pico R _II_ e 3 e 4 ms após o pico R _II_ mudou no Pós-Ex 5. As causas exatas dessas alterações não são claras. Como mostramos, no momento de 3 ms após R _II_ -pico, a segunda inversão das distribuições de potencial nos BSPMs ainda continuava no Pré-Ex, ao passo que já estava completa no Pós-Ex 5. Portanto, a diminuição da amplitude dos extremos negativos do BSPM 3 e 4 ms após o pico R _II_ no Pós-Ex 5 em comparação com o Pré-Ex pode estar associada a uma conclusão precoce da segunda inversão de distribuições dos potenciais nos BSPMs.

Em resumo, de acordo com os resultados, este é o primeiro estudo a mostrar as distribuições potenciais de superfície corporal durante a despolarização ventricular em ratos após exercício exaustivo agudo. Sugerimos que as mudanças transitórias nas características temporais e de amplitude dos BSPMs observadas em ratos, após corrida em esteira até a exaustão, foram fisiológicas e refletiram o comportamento elétrico do coração em exercício físico extenuante.

O presente estudo tem limitações. Primeiro, nossos resultados são restritos apenas a ratos machos adultos jovens e não podem ser aplicados diretamente à população de ratos de laboratório como um todo. Em segundo lugar, no presente estudo, o registro da atividade elétrica cardíaca foi realizado em animais anestesiados com zoletil. Embora tenha sido demonstrado que o zoletil tem um efeito cardiovascular mínimo, ^41^ a influência desse anestésico nos dados obtidos não pode ser completamente excluída.

## Conclusão

Em conclusão, nossos dados mostraram que o exercício agudo em esteira até a exaustão não alterou o padrão espacial das distribuições de potenciais de superfície corporal durante a despolarização ventricular, mas induziu a diminuição da duração da fase média e da duração total da despolarização ventricular, bem como as mudanças na amplitude do extremo negativo dos BSPMs durante a despolarização ventricular em ratos.
