# Supplementary material for: Mapeamento Potencial de Superfície Corporal durante a Despolarização Ventricular em Ratos Após Exercício Exaustivo Agudo
Source: Arq Bras Cardiol. 2022 Sep 6;119(5):766–75. [Article in Portuguese] doi: 10.36660/abc.20211058 (PMC9750213; doi:10.36660/abc.20211058)
Supplement: Translation

# Translation: Body Surface Potential Mapping during Ventricular Depolarization in Rats after Acute Exhaustive Exercise

**Authors:** Alexey G. Ivonin, Svetlana L. Smirnova, Irina M. Roshchevskaya

**Affiliations:** 1 Russian Federation Department of Comparative Cardiology – Komi Scientific Centre of the Ural Branch of the Russian Academy of Sciences, Syktyvkar – Russian Federation; 2 Russian Federation Laboratory of Pharmacological Screening – Research Zakusov Institute of Pharmacology, Moscow – Russian Federation

**Keywords:** Rats, Heart Ventricles, Myocardium, Exercises, Acute, Body Surface Potential Mapping

## Abstract

**Background:**

Exhaustive physical exercise can cause substantial changes in the electrical properties of the myocardium.

**Objective:**

To evaluate, using body surface potential mapping, the electrical activity of the heart in rats during ventricular depolarization after acute exhaustive exercise.

**Methods:**

Twelve-week-old male rats were submitted to acute treadmill exercise at 36 m/min until exhaustion. Unipolar electrocardiograms (ECGs) from the torso surface were recorded in zoletil-anesthetized rats three to five days before (Pre-Ex), 5 and 10 minutes after exhaustive exercise (Post-Ex 5 and Post-Ex 10, respectively) simultaneously with ECGs in limb leads. The instantaneous body surface potential maps (BSPMs) were analyzed during ventricular depolarization. P values <0.05 were considered statistically significant.

**Results:**

Compared with Pre-Ex, an early completion of the second inversion of potential distributions, an early completion of ventricular depolarization, as well as a decrease in the duration of the middle phase and the total duration of ventricular depolarization on BSPMs were revealed at Post-Ex 5. Also, compared with Pre-Ex, an increase in the amplitude of negative BSPM extremum at the R-wave peak on the ECG in lead II (R_II_-peak) and a decrease in the amplitude of negative BSPM extremum at 3 and 4 ms after R_II_-peak were showed at Post-Ex 5. At Post-Ex 10, parameters of BSPMs did not differ from those at Pre-Ex.

**Conclusion:**

In rats, acute exhaustive exercise causes reversible changes in the temporal and amplitude characteristics of BSPMs during ventricular depolarization, most likely related to alterations in the excitation of the main mass of the ventricular myocardium.

## Introduction

The beneficial effects of regular physical exercise on public health, including reduced risk of cardiovascular diseases, have been well established.^1 , 2^ However, excessive exercise can cause damage to the body, especially the heart. A number of studies have demonstrated a transient deterioration in cardiac function after acute bouts of prolonged endurance exercise in apparently healthy individuals.^3 , 4^ Excessive physical exercise may be a trigger for malignant ventricular arrhythmias, acute myocardial infarction and sudden cardiac death in susceptible persons.^5^

Research on the impact of exhaustive exercise on the cardiovascular system is essential. But, in humans, it is difficult to objectively determine exhaustion under laboratory conditions, since it is measured by the inability of the subject to maintain the exercise regimen by volitional effort.^6 , 7^ In this regard, the advantage of model animals (e.g., rats, mice) in evaluating physiological responses to exhaustive exercises is that there are objective criteria for the definition of exhaustion, such as the inability of an animal to continue running despite the external stimuli during a treadmill running test^8 , 9^ or sinking to the bottom of the pool during a forced swimming test.^10^

Numerous studies in laboratory animals have shown myocardial tissue damage and cardiac function impairment associated with cardiomyocyte apoptosis, oxidative stress and inflammatory responses after acute bouts of exhaustive exercise.^11 - 13^ It has been reported that acute exhaustive exercise causes changes in electrocardiographic patterns in rats, suggesting ventricular depolarization alterations.^14 , 15^

Body surface potential mapping based on a simultaneous register of cardiac-generated potentials, from multiple sites, over the entire torso surface, provides more physiological and diagnostic information about electrical events in the myocardium, compared with conventional electrocardiography.^16 - 18^ By using body surface potential mapping, the present study aimed to evaluate the electrical activity of the heart in laboratory rats during ventricular depolarization after acute exhaustive exercise.

## Methods

### Experimental animals

Twelve-week-old male outbred albino rats (n=24), weighing between 200 and 300 g, were purchased from the Stolbovaya Branch of the Scientific Center for Biomedical Technologies of the Federal Medical-Biological Agency (Russian Federation). The rats were housed in group cages in a temperature-controlled room (22 ± 2°C) under a 12-h light/dark cycle and fed standard commercial rodent chow and water *ad libitum* . All experimental procedures and protocols were carried out in accordance with the Guide for the Care and Use of Laboratory Animals published by the National Institutes of Health (NIH Publication No. 85-23, revised 1996) and approved by the Ethics Committee of the Komi Science Centre of the Ural Branch of the Russian Academy of Sciences (Syktyvkar, Russian Federation).

### Exercise protocol

A motorized rodent treadmill (Panlab / Harvard Apparatus, Spain) was used to create acute exhaustive exercise for rats. Mild electrical pulses (0.5 mA) from the electrified grid on the rear of the treadmill motivated the animals to exercise. Before experimentation, the rats were accustomed to treadmill exercise for three consecutive days (10 min/day, 12-36 m/min, 0° incline). Only certain rats, capable of running in an adaptive mode, were selected for further study. On the day of the exercise test, the rats were running at a speed of 36 m/min and 0° incline until exhaustion. Exhaustion was defined as the point at which the rat could not run anymore, despite being pushed against the shock grid by the moving treadmill belt.^8^ The running time to exhaustion was computed using Sedacom version 2.0 software (Panlab / Harvard Apparatus, Spain).

### Conventional electrocardiography and body surface potential mapping

The registration of heart electrical activity was performed three to five days before (Pre-Ex), 5 and 10 minutes after (Post-Ex 5 and Post-Ex 10, respectively) acute exhaustive exercise. The rats were anesthetized with Zoletil (tiletamine / zolazepam combination, Virbac, France) at a dose of 3.5 mg / 100 g, i.m. and placed in the supine position on a heating pad to maintain body temperature approximately at 37°C. Unipolar electrocardiograms (ECGs) were recorded from subcutaneous needle electrodes, evenly distributed around the torso from the level of the cervicothoracic junction to the inferior rib margins ( Figure 1 ). A total of 64 electrodes at Pre-Ex and 32 ones at Post-Ex 5 and Post-Ex 10 were used. Simultaneously with unipolar ECGs from the surface of the torso, ECGs were recorded in bipolar limb leads. The Wilson central terminal served as a reference for the unipolar torso leads. The data were acquired using a multichannel computer system (bandwidth of 0.05-1000 Hz, sampling rate of 4000 Hz, and accuracy of 16 bits). The rats had a period of three to five days to recover from anesthesia prior to exhaustive exercise. To register post-exercise cardiac potentials, the rats were anesthetized immediately after exhaustion.

**Figure 1:** Positions of electrodes on the rat’s body surface using 64 unipolar torso leads. Four rows of needle electrodes were placed on the ventral, and four rows on the dorsal surface of the body (eight electrodes in each row). When using 32 unipolar torso leads, the number of electrode rows on the ventral and dorsal body surfaces decreased twice.

The duration of R-R intervals and ventricular depolarization parameters (QRS duration, R- and S-wave duration and amplitude, the sum of R- and S-wave amplitudes) were analyzed on the ECG in the limb lead II (ECG_II_). The R-R intervals were used to compute the heart rate (HR). Based on unipolar ECGs, instantaneous body surface equipotential (isopotential) maps were constructed, reflecting the distribution of cardiac potentials in each instant of ventricular depolarization on a flat pattern of the chest surface aligned to a rectangular plane. The body surface potential maps (BSPMs) analyzed the spatial location and shifting trajectories of the areas and extrema of positive and negative potentials on the thorax surface, the amplitudes of extrema in sequential time instants during ventricular depolarization, the maximum amplitudes of extrema for the whole ventricular depolarization, and the time when the extrema reached maximum amplitudes. Temporal characteristics of the BSPMs were presented in ms, relative to the peak of R_II_-wave (up to the R_II_-peak – with a minus sign).

According to the spatial-temporal dynamics of body surface potential distribution, the following phases of ventricular depolarization were differentiated:^19^

1. Initial phase – from the formation of a cardiac potential distribution pattern, corresponding to ventricular depolarization, to the completion of the first inversion of positive and negative potential areas.
2. Middle phase – from completion of the first inversion to completion of the second inversion of potential areas.
3. Terminal phase – from the completion of the second inversion of potential areas to the disappearance of potential distribution pattern corresponding to ventricular depolarization.

For each rat, characteristics of ECG_II_and BSPMs were determined from the three to five beats at Pre-Ex, Post-Ex 5, and Post-Ex 10.

### Statistical analysis

The statistical analysis was performed using a Statistica software package (version 10.0, StatSoft, Tulsa, OK, USA). Continuous data normality was checked using the Shapiro-Wilk test. Normally distributed variables were expressed as mean ± standard deviation, and non-normally distributed variables were presented as median, first and third quartiles. For the data with normal distribution, the repeated measures analysis of variance (ANOVA) was used, followed by Dunnett’s multiple comparison test as a *post-hoc* analysis. When the data were non-normally distributed, the non-parametric Friedman test, followed by Wilcoxon’s test with Bonferroni adjustment, was performed. A statistical significance was established at an alpha level of 0.05, except for Wilcoxon’s test, in which the alpha level was adjusted to 0.025 (according to the number of pairwise comparisons) to avoid a type I error. The sample size was determined by convenience. Data^20^ have been taken into account that up to 10% of rats from commercial vendors refuse to run on a treadmill and should be eliminated from exercise studies.

## Results

### Exercise performance

Of 24 rats used to get familiarized with running sessions, 20 were selected for the acute exhaustive exercise according to their running ability. In these rats, the running time to exhaustion was 19.5 ± 5.6 min.

### Conventional ECG parameters

Table 1 shows the ECG_II_findings. There were no statistically significant changes in HR values after exhaustive treadmill exercise. Compared with those at Pre-Ex, the QRS_II_duration and R_II_-wave duration were lower at Post-Ex 5. Compared with those at Pre-Ex, the S_II_-wave amplitude and the sum of R_II_- and S_II_-wave amplitudes were higher at Post-Ex 5 and Post-Ex 10. There were no significant changes in either the S_II_-wave duration or the R_II_-wave amplitude after exhaustive treadmill running.

**Table 1:** ECGII parameters before and after acute exhaustive treadmill exercise

	Pre-Ex	Post-Ex 5	Post-Ex 10
HR, bpm	480.3 ± 23.3	484.5 ± 25.2	490.7 ± 28.8
QRS_II_duration, ms	16.0 ± 1.1	15.3 ± 1.2*	15.8 ± 1.7
R_II_-wave duration, ms	9.7 ± 1.0	9.1 ± 0.9*	9.4 ± 1.0
S_II_-wave duration, ms	6.3 ± 1.4	6.2 ± 1.5	6.3 ± 1.7
R_II_-wave amplitude, mV	0.62 ± 0.16	0.65 ± 0.15	0.63 ± 0.14
S_II_-wave amplitude, mV	-0.28 ± 0.15	-0.35 ± 0.18*	-0.35 ± 0. 19*
Sum of RII- and SII-wave amplitudes, mV	0.90 ± 0.19	0.99 ± 0.20*	0.98±0.20*

Data are expressed as mean ± standard deviation (n = 20). Pre-Ex: before exhaustive exercise; Post-Ex 5: 5 minutes after exhaustive exercise; Post-Ex 10: 10 minutes after exhaustive exercise. HR: heart rate. Repeated measures ANOVA and Dunnett’s post-hoc test; *p < 0.05 vs. Pre-Ex.

### Spatial pattern of BSPMs

Before exhaustive exercise, the body surface potential distribution pattern, corresponding to the onset of ventricular depolarization, was observed prior to the appearance of the QRS complex on ECG_II_( Figure 2 , Pre-Ex, instant –9 ms). In this case, the area of the positive cardiac potentials covered the cranial part of the ventral chest and the entire dorsal chest, with the positive extremum being located mainly in the cranial third of the left-lateral chest. The area of negative potentials, and the negative extremum, were located in the caudal part of the ventral chest. During the upslope of R_II_-wave, the first inversion of potential distributions occurred on BSPMs, and as a result, the positive and negative areas changed their relative positions ( Figure 2 , Pre-Ex, instant –5.5 ms). By the completion of the first inversion, the positive extremum was located caudally on the ventral chest, and the negative extremum was located cranially, more frequently on the back. In the instant of the R_II_-wave peak, the location of areas and extrema on BSPMs remained essentially unchanged ( Figure 2 , Pre-Ex, instant 0 ms). During the downslope of R_II_- and S_II_-waves, the second inversion of potential distributions was observed on the BSPMs, and as a consequence, the area of negative potentials was located in the caudal part of the chest, left laterally or ventrally, while the area of positive potentials occupied the remaining chest surface ( Figure 2 , Pre-Ex, instant 4.0 ms). By the completion of the second inversion, the positive extremum shifted cranially and right laterally, with the negative extremum being located caudally on the left lateral chest. During the transition from S_II_-wave to T_II_-wave, a pattern was recorded on the BSPMs of an unstable position of the positive and negative areas, which indicated the completion of the ventricular depolarization ( Figure 2 , Pre-Ex, instant 5.5 ms). After exhaustive exercise, spatial potential distributions during ventricular depolarization were quite similar to those at Pre-Ex ( Figure 2 , Post-Ex 5 and Post-Ex 10).

**Figure 2:** Body surface equipotential maps during ventricular depolarization before and after acute exhaustive treadmill exercise in the same rat. The areas of positive (filled) and negative potentials are shown. The signs, plus and minus, show the location of the positive and negative extrema, respectively. Below each map, the time relative to the RII-wave peak and the amplitudes of the positive and negative extrema are shown. Near to each map, ECGII is shown with a time marker (vertical line). The isopotential contour spacing is 0.2 mV. In each map, the left part represents the ventral body surface, and the right represents the dorsal body surface. Pre-Ex: before exhaustive exercise; Post-Ex 5: 5 minutes after exhaustive exercise; Post-Ex 10: 10 minutes after exhaustive exercise.

### Temporal characteristics of BSPMs

There were no significant changes in either the time of ventricular depolarization onset or the time of the first inversion completion of potential distributions on BSPMs after exhaustive treadmill exercise ( Figure 3 ). Compared with that at Pre-Ex, the second inversion of potential distributions on BSPMs was completed significantly earlier at Post-Ex 5 ( Figure 3 ). In addition, compared with that at Pre-Ex, ventricular depolarization on BSPMs was completed significantly earlier at Post-Ex 5 ( Figure 3 ).

**Figure 3:** Temporal parameters of BSPMs during ventricular depolarization, before and after acute exhaustive treadmill exercise. The time is shown relative to the RII-wave peak. Data are expressed as mean ± standard deviation (n = 20). Pre-Ex: before exhaustive exercise; Post-Ex 5: 5 minutes after exhaustive exercise; Post-Ex 10: 10 minutes after exhaustive exercise. Repeated measures ANOVA and Dunnett’s post-hoc test; *p < 0.05 vs. Pre-Ex.

No significant changes in the duration of the initial and the terminal phases of ventricular depolarization were observed on the BSPMs after exhaustive treadmill running ( Figure 4 ). Compared with those at Pre-Ex, the duration of the middle phase and the total duration of ventricular depolarization decreased at Post-Ex 5 ( Figure 4 ).

**Figure 4:** Duration of individual phases and total duration of ventricular depolarization on BSPMs before and after acute exhaustive treadmill exercise. Data are expressed as mean ± standard deviation (n = 20). PreEx: before exhaustive exercise; Post-Ex 5: 5 minutes after exhaustive exercise; Post-Ex 10: 10 minutes after exhaustive exercise. Repeated measures ANOVA and Dunnett’s post-hoc test; *p < 0.05 vs. Pre-Ex.

### Characteristics of BSPM extrema

During ventricular depolarization, the amplitudes of positive and negative BSPM extrema pre- and post-exhaustive treadmill exercise progressively increased, reaching maximum values near the peak of the R_II_-wave, then decreased ( Figure 2 , Table 2 ). There were no significant changes in either the maximum amplitudes of BSPM extrema during ventricular depolarization, or the time when extrema reached maximum amplitudes after acute exhaustive exercise ( Figure 5 ).

**Table 2:** Amplitudes of BSPM extrema at different time moments during ventricular depolarization before and after acute exhaustive treadmill exercise

		Time moment, ms									
		-5	-4	-3	-2	-1	0	1	2	3	4
Positive extremum, mV	Pre-Ex	0.19 (0.06; 0.31)	0.44 (0.19; 0.54)	0.67 (0.41; 0.81)	0.89 (0.80; 1.09)	1.06 (0.90; 1.20)	0.92 (0.79; 1.16)	0.74 (0.54; 0.91)	0.45 (0.17; 0.79)	0.41 (0.29; 0.63)	0.4 (0.33; 0.52)
	Post-Ex 5	0.14 (0.05; 0.24)	0.34 (0.19; 0.46)	0.57 (0.43; 0.73)	0.88 (0.67; 1.05)	1.02 (0.78; 1.20)	0.95 (0.77; 1.15)	0.76 (0.39; 0.9)	0.33 (0.22; 0.59)	0.44 (0.19; 0.59)	0.34 (0.20; 0.46)
	Post -Ex 10	0.19 (0.06; 0.28)	0.41 (0.19; 0.50)	0.67 (0.44; 0.77)	0.84 (0.56;1.02)	0.95 (0.75; 1.16)	0.95 (0.76; 1.15)	0.61 (0.28; 0.91)	0.39 (0.18; 0.57)	0.41 (0.21; 0.58)	0.4 (0.2; 0.53)
Negative extremum, mV	Pre-Ex	-0.14 (-0.11; -0.21)	-0.28 (-0.16; -0.35)	-0.40 (-0.26; -0.52)	-0.58 (-0.38; -0.64)	-0.64 (-0.51; -0.73)	-0.71 (-0.62; -0.85)	-0.58 (-0.49; -0.74)	-0.42 (-0.29; -0.56)	-0.28 (-0.20; -0.40)	-0.16 (-0.09; -0.27)
	Post -Ex 5	-0.14 (-0.07; -0.19)	-0.26 (-0.18; -0.35)	-0.42 (-0.27; -0.50)	-0.57 (-0.41;-0.67)	-0.65 (-0.58; -0.78)	-0.77 (-0.68; -0.92)*	-0.65 (-0.44; -0.78)	-0.39 (-0.22; -0.44)	-0.21 (-0.14; -0.30)*	-0.08 (-0.05; -0.189)*
	Post -Ex 10	-0.16 (-0.08; -0.20)	-0.29 (-0.17; -0.35)	-0.45 (-0.30; -0.53)	-0.60 (-0.40; -0.69)	-0.66 (-0.55; -0.75)	-0.75 (-0.60; -0.90)	-0.67 (-0.45; -0.73)	-0.39 (-0.20; -0.46)	-0.23 (-0.13; -0.36)	-0.12 (-0.06; -0.23)

The time is shown relative to the RII-wave peak. Data are expressed as median, first and third quartiles (n = 20). Pre-Ex: before exhaustive exercise; Post-Ex 5: 5 minutes after exhaustive exercise; Post-Ex 10: 10 minutes after exhaustive exercise. Wilcoxon test with Bonferroni adjustment; *p < 0.025 vs. Pre-Ex at the same time.

**Figure 5:** Maximum amplitudes of positive (A) and negative (B) extrema on BSPMs during ventricular depolarization, and the time when positive and negative extrema reached maximum amplitudes (C and D, respectively) before and after acute exhaustive treadmill exercise. The time is shown relative to the RIIwave peak. Data are expressed as median, first and third quartiles, minimum and maximum (n = 20). PreEx: before exhaustive exercise; Post-Ex 5: 5 minutes after exhaustive exercise; Post-Ex 10: 10 minutes after exhaustive exercise. Friedman test, p > 0.05.

The amplitudes of BSPM extrema at different moments during ventricular depolarization are shown in Table 2 . Due to the individual variations in the onset and completion times of ventricular depolarization on BSPMs, the time range corresponding to the depolarization of ventricles in all studied animals (i.e. from -5 to 4 ms relative to R_II_-peak) was analyzed. At each analyzed moment of the chosen time range, no significant changes in the amplitude of positive BSPM extremum were observed after exhaustive treadmill exercise. At the R_II_-peak, the amplitude of negative BSPM extremum at Post-Ex 5 was higher compared with that at Pre-Ex ( Table 2 , instant 0 ms). Additionally, 3 and 4 ms after R_II_-peak, the amplitudes of negative BSPM extremum at Post-Ex 5 were lower compared with those at Pre-Ex ( Table 2 , moments 3 and 4 ms).

## Discussion

In the present study, the electrical activity of the heart was evaluated during ventricular depolarization after acute exhaustive treadmill exercise in previously untrained rats, by using body surface potential mapping in combination with conventional electrocardiography.

The immediate cardiovascular response to forced treadmill exercise in rodents is an increase in HR,^21 , 22^ ensuring an increase in cardiac output. In Wistar rats, the HR acceleration, during treadmill exercise, is predominantly determined by the cardiac autonomic nerve activity, especially by an augmentation in sympathetic outflow.^23^ The decrease in HR, after physical exercise cessation, is likely the manifestation of vagal reactivation.^24^ Previous studies on untrained rats have demonstrated the decline in HR to the initial level at 10 minutes after acute exhaustive treadmill running.^25^ In the present study, in both situations after exhaustive exercise (Post-Ex 5 and Post-Ex 10), HR values were nearly equal to that at baseline (Pre-Ex). In our opinion, these results could be explained by a decrease in HR to the pre-exercise level within the first 5 minutes following running until exhaustion.

In healthy human subjects, the ECG response to physical exercise consists of either a decrease in the QRS duration or no changes in QRS duration.^26 , 27^ The shortening of the QRS duration with exercise in healthy individuals is attributed to enhanced intraventricular conduction velocity, due to increased adrenergic tone.^26^ Meanwhile, QRS prolongation with exercise is considered an abnormal ECG finding and might be used as a marker of myocardial ischemia.^28^ The significant prolongation of the duration of QRS, accompanied by the impairment of the cardiac functional capacity, was observed in healthy Sprague-Dawley rats, which were submitted to acute exhaustive treadmill exercise, compared with their sedentary control.^15^ We found a decrease in the QRS_II_duration and R_II_-wave duration at Post-Ex compared with those at Pre-Ex, while at Post-Ex 10 these parameters returned to near Pre-Ex level. It is generally accepted that, in humans, the R-wave in limb leads of ECG represents the depolarization of the left and right ventricles, and S-wave reflects predominantly the excitation of the basal parts of ventricles.^29^ The genesis of R- and S-waves on ECGs in rats is similar to that in humans.^30^ Thus, our results suggested that the changes in QRS duration after acute exhaustive exercise in rats were related to a transient decrease in the excitation duration in most of the ventricular myocardium.

In apparently healthy humans, a decrease in the R-wave amplitude, with or without an increase in the S-wave amplitude, is observed on ECGs at maximal exercise.^31 , 32^ The reduction in the R-wave amplitude, with strenuous exercise, is attributed to a decrease in the ventricular end-diastolic volume,^27^ as well as to changes in cardiac electrical activation,^31^ while the increase in S-wave amplitude is assumed to be related to electrical axis shifts or ventricular conduction alterations.^33^ In this study, the R_II_-wave amplitude did not alter after exhaustive treadmill running. However, compared with Pre-Ex, the amplitude of S_II_-wave increased at Post-Ex 5 and Post-Ex 10. Since the sum of the R_II_- and S_II_-wave amplitudes also increased at Post-Ex 5 and Post-Ex 10, variations in S_II_-wave amplitude following exhaustive exercise were probably not the result of a QRS axis deviation. Because of the lack of an isoelectric ST-segment in rats, the increase in the S-wave amplitude on rat ECGs is regarded as the ST-segment depression and is interpreted as a sign of myocardial ischemia.^34 , 35^ So, in the present study, the increase in the amplitude of S_II_-wave in exhausted rats may be caused by exercise-induced ischemic alterations in heart ventricles. Nonetheless, further studies are needed to confirm this assumption.

Changes in the spatial distribution of cardiac potentials on the body surface, during ventricular depolarization, are indicators of alterations in the direction of excitation wavefronts in the heart ventricles.^16 , 36^ Miller et al.^37^ observed significant changes in the migration trajectory of negative extremum, as well as the appearance of additional anterior positive extrema on BSPMs during the QRS complex at maximal exercise in healthy subjects, which they considered to be due to a delay in the activation of the left ventricular free wall, relative to the onset of excitation in the right ventricle and intraventricular septum.

Takala et al.^38^ disclosed no changes in spatial patterns of QRS-time BSPMs after maximal exercise in healthy individuals. In the present study, spatial BSPM patterns during ventricular depolarization were similar to those previously observed in healthy Wistar rats.^39^ The locations of areas and extrema, and their dynamics on BSPMs at Post-Ex 5 and Post-Ex 10 were almost identical to those at Pre-Ex, which suggested that the main direction of the activation wave in rat ventricles did not change greatly with acute exhaustive treadmill exercise.

In rats, the first inversion of potential distributions on the BSPMs, during ventricular depolarization, is caused by a breakthrough of the excitation wave on the subepicardium of both the base of the right ventricle and the apex of the left ventricle, and the second inversion resulted from the change in the direction of the activation wave towards the base of the left ventricle and the excretory cone of the aorta.^19^ During the initial phase of ventricular depolarization on BSPMs, the excitation wave, in rats, spreads through the conducting system and then moves through the myocardium in an endo-epicardial direction. During the middle and terminal phases of ventricular depolarization on BSPMs, there is an excitation of the main mass of the ventricular myocardium, and the base of the left ventricle, respectively.^19^ In this study, at Post-Ex 5, in comparison with Pre-Ex, an earlier completion of the second inversion of potential distributions, an earlier completion of ventricular depolarization, as well as a decrease in the duration of the middle phase and the overall duration of ventricular depolarization were demonstrated on BSPMs, which appears to have resulted from a decrease in the activation duration of the main mass of the ventricular myocardium. At Post-Ex 10, temporal parameters of BSPMs did not differ from those at Pre-Ex. Thus, in the present study, the changes in the temporal BSPM characteristics, subsequent to acute exhaustive treadmill exercise, were caused by a reversible reduction in the duration of the middle phase of ventricular depolarization, while the durations of the initial and terminal phases remained essentially unchanged. Compared with conventional electrocardiography, body surface potential mapping allowed a more accurate identification of the stage of ventricular depolarization, the duration of which was considerably altered as a result of exhaustive treadmill exercise in rats.

Regarding the effect of strenuous exercise on the amplitude of BSPM parameters, Mirvis^40^ described a reduction in the amplitude of positive extremum and an increase in the amplitude of negative extremum on isopotential maps of the anterior surface of the thorax at different points of the QRS complex during submaximal exercise in healthy subjects. Other authors^37^ revealed the decrease in the maximum amplitude of the positive BSPM extremum during QRS, with maximal exercise in healthy volunteers, which they surmised was a result from the changes in activation wavefronts in the left ventricular wall. In our study, the maximum amplitudes of positive and negative BSPM extrema during ventricular depolarization, and the time when the extrema reached maximum amplitudes, did not alter significantly after exhaustive treadmill exercise. Meanwhile, compared with those at Pre-Ex, the amplitude of negative BSPM extremum in the instant of R_II_-peak and 3 and 4 ms after R_II_-peak changed at Post-Ex 5. The exact causes of these changes are unclear. As we have shown, in the moment of 3 ms after R_II_-peak, the second inversion of potential distributions on BSPMs was still continuing at Pre-Ex, whereas it was already completed at Post-Ex 5. Therefore, the decrease in amplitude of the negative BSPM extremum 3 and 4 ms after R_II_-peak at Post-Ex 5 as compared with those at Pre-Ex may be associated with an earlier completion of the second inversion of potential distributions on BSPMs.

In summary, according to the results, this is the first study to show the body surface potential distributions during ventricular depolarization following acute exhaustive exercise in rats. We suggest that the transient changes in temporal and amplitude characteristics of BSPMs observed in rats, after treadmill running until exhaustion, were physiological and reflected the electrical behavior of the heart at strenuous physical exercise.

The present study has limitations. First, our results are restricted only to young adult male rats and cannot be applied directly to the population of laboratory rats as a whole. Second, in the current study, the recording of the heart electrical activity was performed in zoletil-anesthetized animals. Although it has been shown that zoletil has a minimal cardiovascular effect,^41^ the influence of this anesthetic on the obtained data cannot be completely excluded.

## Conclusion

In conclusion, our data showed that acute treadmill exercise until exhaustion did not alter the spatial pattern of body surface potential distributions during ventricular depolarization but induced the decrease in the duration of the middle phase, and the total duration of ventricular depolarization, as well as the changes in the amplitude of negative extremum of BSPMs during ventricular depolarization in rats.
